# Ethical, Medicolegal, and Organisational Pressures Shape Patient Safety at Hospital Interfaces: A Qualitative Study from Romania

**DOI:** 10.3390/healthcare14111542

**Published:** 2026-06-01

**Authors:** Andrada-Georgiana Nacu, Dan-Alexandru Constantin, Liliana Marcela Rogozea

**Affiliations:** Department of Fundamental, Prophylactic and Clinical Sciences, Faculty of Medicine, Transilvania University of Brasov, 500019 Brasov, Romania; englober@icloud.com (D.-A.C.); r_liliana@yahoo.com (L.M.R.)

**Keywords:** patient safety, qualitative research, informed consent, incident reporting, speaking up, medical documentation

## Abstract

Background and Objectives: Patient safety at hospital interfaces is shaped by organisational fragility, ethical obligations, and anticipated legal exposure. Reporting, disclosure, and speaking up have been studied separately, yet the way these pressures converge in ordinary hospital work remains insufficiently described. Materials and Methods: We conducted a qualitative study in a public hospital in Romania using semi-structured episodic interviews and the critical incident technique. Twelve clinicians participated: six nurses and six physicians working in intensive care, emergency medicine, general surgery, paediatrics, oncology day care, anaesthesia, obstetrics, and internal medicine/cardiology. Interviews were audio-recorded, transcribed verbatim in Romanian, anonymised, and analysed with the framework method from a critical realist perspective. A secondary cross-case coding of all 12 episodes was used for descriptive analytic displays. Results: Four mechanisms organised the material. First, local stop rules and cross-checks created temporary stability at fragile interfaces such as high-alert medication, patient identification, specimen labelling, and transfer documentation. Second, consent and confidentiality were repeatedly compressed by urgency, compromised capacity, public space, and family pressure; legitimacy depended on explicit reasoning rather than documentary completion alone. Third, speaking-up and near-miss reporting were governed by protocol-backed legitimacy, leader response, and the informal cost of interruption. Formal incident reporting was present in one episode, partial in one, and absent in 10. Fourth, documentation and disclosure redistributed accountability. Notes that recorded reasoning supported continuity of care, whereas protective opacity concealed near misses, infrastructural weakness, and interactional pressure. Documentation or disclosure pressure appeared in all 12 episodes. Conclusions: Safety in everyday hospital work was assembled through local barriers, moral triage, and selective visibility. Interface redesign, protected near-miss reporting, psychologically safe escalation, and structured support for urgent consent and post-incident communication would make transparent safety work more sustainable. Trustworthiness was strengthened through reflexive memoing by the physician-interviewer, an audit trail of coding decisions, comparison across professional groups, active attention to negative cases, and iterative assessment of meaning saturation at the level of explanatory mechanisms.

## 1. Introduction

Patient harm remains a persistent feature of hospital care, and contemporary safety programmes increasingly acknowledge that preventable harm is generated through organisational design, communication, workflow, and culture as much as through isolated technical error [1,2]. Research in resilient health care has clarified why the distinction between work as imagined and work as done matters for safe performance: staff routinely hold systems together through adaptation, escalation, checking and compromise [3,4]. That line of work has been especially productive in settings marked by volatility, multitasking and weak information flow.

Hospital interfaces expose this dynamic with unusual clarity. Medication preparation, specimen handling, patient identification, handover, urgent consent, and communication with relatives bring together clinical judgement, time pressure, documentation, and accountability in the same moment [5,6,7]. Speaking-up studies have shown that clinicians calculate the interpersonal cost of interruption before they act [8,9]. Incident reporting studies have shown that near misses often disappear into informal correction when reporting systems feel punitive, bureaucratic, or clinically unrewarding [10,11]. Disclosure research has traced the tension between transparency, empathy, and anticipated liability after harm [12,13].

Compared with previous qualitative work that has often examined speaking up, near-miss reporting, consent, confidentiality, documentation, or disclosure as separate domains, the novelty of this study lies in following how these pressures converge within the same ordinary safety episodes. We therefore treat the hospital interface as an analytic site where bedside checking, ethical reasoning, documentation, reporting decisions, and anticipated liability become mutually entangled before harm is either prevented, disclosed, or rendered institutionally invisible.

What remains less well specified is the mechanism by which these strands meet in routine hospital work before catastrophic harm occurs. In practice, ethical duties, organisational fragility, and medicolegal anticipation are rarely separate domains. A compressed consent discussion, a delayed administration, an impersonal note, or an omitted near-miss label may each represent a local attempt to preserve safety under conditions that narrow what can be said, recorded, or escalated. This study explored how nurses and physicians working in Romanian hospital settings identified, prioritised, and managed safety risks in everyday practice. The analytic aim was explanatory rather than merely descriptive: to identify recurrent mechanisms linking conditions, local adaptations, decisions, and consequences across clinical episodes.

## 2. Materials and Methods

### 2.1. Setting and Design

This qualitative study was undertaken within the qualitative strand of an explanatory sequential mixed-methods programme on patient safety in hospital care in Romania. This article reports the qualitative component only. A critical realist orientation informed the design, on the premise that organisational arrangements, professional hierarchies, infrastructural fragility, and medicolegal pressures have real effects, even though they are accessed through situated accounts [14,15].

Fieldwork was conducted in a public hospital in Romania between 1 February 2026 and 10 March 2026. Clinical areas were selected because emergency flow, invasive procedures, high-alert medication, complex handovers, or intense interaction with relatives made safety dilemmas especially visible.

### 2.2. Sampling and Participants

Purposive maximum-variation sampling was used to capture diversity in profession, seniority, and setting. Eligible participants were nurses and physicians with current clinical activity in the included units and at least six months of experience in their clinical area. Twelve clinicians participated: six nurses and six physicians. Their settings included intensive care, general surgery, emergency medicine, paediatrics, oncology day care, anaesthesia, obstetrics, and internal medicine/cardiology (Table 1). Recruitment stopped when additional interviews no longer generated new mechanistic propositions across professions and settings.

Participants were approached through a neutral invitation after institutional access had been agreed, and interviews were scheduled individually in settings where confidentiality could be protected. Recruitment and preliminary analysis proceeded iteratively: early interviews were summarised before later interviews, and subsequent sampling was used to check whether the emerging mechanisms held across professional role and clinical setting. The retained study log documents the 12 completed interviews; a separate denominator log of all staff invited and all refusals was not maintained, so exact invitation and refusal numbers cannot be reconstructed without introducing unsupported data.

Non-clinical staff, staff whose primary responsibilities were administrative or managerial rather than bedside clinical care, clinicians with fewer than six months of experience in the relevant clinical area, and clinicians in a direct supervisory or dependent professional relationship with the interviewer were not eligible. Interviews were also not conducted in circumstances where confidentiality of the conversation could not be protected.

Saturation was assessed primarily as meaning saturation rather than simple code saturation. After each interview, the team considered whether the corpus had produced new explanatory relations between conditions, local adaptations, decision points, and consequences, or whether later accounts mainly added further examples of already identified mechanisms. Recruitment stopped when the last interviews enriched contrastive detail but did not generate a new mechanism across the nurse and physician groups.

### 2.3. Data Collection

Interviews were conducted by a physician in residency training with university affiliation and no direct supervisory relationship with participants. Recruitment and interviewing were framed explicitly as non-evaluative. Neutral invitations were used, interviews were scheduled individually, and participants were asked not to mention patient or colleague identifiers. Each participant took part in one interview.

Semi-structured episodic interviews were organised around a recent incident, near miss, or safety-critical episode and used the critical incident technique to reconstruct chronology, decision points, escalation, documentation, reporting, and communication [14]. The guide covered seven analytic domains: work as done, informed consent, confidentiality, interprofessional coordination, speaking up, incident reporting, and the distinction between clinical and defensive documentation. Interviews were audio-recorded, transcribed verbatim in Romanian, and anonymised. Quotations were translated into English by the authors. The guide was developed from the study questions, the preceding quantitative strand, and the literature on resilient health care, speaking up, incident reporting, informed consent, confidentiality, documentation, and disclosure. It was tested in two preliminary interviews to increase episodic density, remove questions that elicited mainly normative answers, calibrate interview length, and standardise transitions between the CIT narrative and the ethical/medicolegal probes.

### 2.4. Analysis

Data were analysed with the framework method [15]. An initial analytic structure derived from the interview guide and from the study focus on ethical, medicolegal, and legislative dimensions of safety. Inductive coding remained open to unanticipated categories. After familiarisation, coding, matrix development, and comparison across cases, the material was synthesised through a Conditions-Adaptations-Decisions-Consequences logic that captured how contextual pressures were translated into observable action. Comparative matrices were built by profession and setting, and negative cases were actively sought in order to refine the explanation.

Initial coding and framework matrixing were undertaken by the interviewing researcher. A formal inter-coder reliability statistic was not calculated because the analysis was interpretive and mechanism-oriented rather than a frequency-coding exercise. Interpretive uncertainties, candidate mechanisms, negative cases, and representative quotations were discussed with the co-authors, who contributed to refining the final coding framework and the Conditions-Adaptations-Decisions-Consequences interpretation.

The Conditions-Adaptations-Decisions-Consequences structure emerged iteratively during analysis rather than being imposed as a fixed template at the start. Early case summaries repeatedly showed a sequence in which contextual pressures, such as time compression, hierarchy, public space, or medicolegal anticipation, led to local adaptations, such as stop rules, compressed explanations, selective documentation, or informal debriefing. Cross-case comparison then clarified the decision points and consequences that linked these adaptations to prevention, opacity, or organisational learning.

For supplementary analytic display, each episode was secondarily coded across six domains: medication or procedural integrity, information integrity or transfer, consent or autonomy, confidentiality or family communication, voice or reporting climate, and documentation or disclosure pressure. These counts were used descriptively only. The six domains were selected because they corresponded to the main interfaces through which the research question became observable: bedside technical integrity, information continuity, patient autonomy, information protection, professional voice, and accountability. These displays were not used as statistical counts, but as a transparent way to show how the same episode could carry several forms of safety pressure simultaneously.

### 2.5. Rigour and Ethics

Rigour was strengthened through variation in sampling, incident-focused interviewing, reflexive memos after interviews, an audit trail of coding decisions, and deliberate attention to negative cases. Reporting followed the consolidated criteria for reporting qualitative research [16]. Participants gave written informed consent. After completion of coding and interpretation, generative AI assistance was used for English-language refinement and document preparation; all analytic content, quotations, and final wording were checked against the source data by the authors.

Reflexivity was treated as an analytic requirement rather than as a disclosure of interviewer characteristics alone. The interviewer was a female physician in residency training with university affiliation, which facilitated understanding of clinical language and workflow but also created a risk of insider assumptions, especially around hierarchy, urgency, and documentation. Reflexive memos after interviews recorded moments where shared professional background may have normalised a workaround or where participants may have framed accounts defensively. During interpretation, attention was paid to the possibility that a physician and a nurse might read the same episode differently: for example, a doctor might frame an urgent consent discussion as clinically necessary compression, whereas a nurse might see the same episode through bedside continuity, patient comprehension, or family pressure.

Trustworthiness was therefore supported by maximum-variation sampling, CIT-based reconstruction of concrete episodes, comparison between nurse and physician accounts, audit-trail notes on coding decisions, active search for negative cases, and author-team discussion of interpretations. The analytic objective was not consensus coding for its own sake, but the refinement of a plausible explanatory account that remained grounded in the interview material.

## 3. Results

### 3.1. Local Stop Rules Stabilised Fragile Interfaces

Participants described safety as a sequence of micro-barriers assembled around known weak points. These weak points clustered at medication preparation, identification, specimen handling, and transfer. In such settings, staff relied on socially recognisable stop rules that could interrupt momentum without requiring a prolonged argument. An intensive care nurse described the logic succinctly: ‘When something does not fit—label versus chart, patient versus wristband, parameters versus dose—I stop and ask for confirmation’ (A01).

Across episodes, the same sequence recurred. A mismatch surfaced at the edge of the process; someone accepted delay, repetition, or friction in exchange for certainty; the episode ended as a prevented error and never entered the incident system. Wrong-patient administration was avoided in the emergency department during wristband downtime because verbal identification interrupted the flow. In internal medicine, laboratory staff recognised that a specimen label and the collection register did not match, prompting withdrawal of the samples before analysis. In cardiology, clarification of the timing of the last anticoagulant dose prevented duplication after transfer. These actions carried an immediate operational cost, yet participants treated that cost as morally and clinically preferable to a silent continuation.

These stop rules were not standardised uniformly across all departments. Some were formal or protocol-backed, such as two-identifier checks, high-alert medication verification, or operative time-out rules; others were local habits sustained by experienced clinicians and particular teams. This uneven institutionalisation was the negative case within an otherwise protective pattern: the same pause that functioned as a barrier in one unit could depend mainly on individual vigilance in another.

An ICU nurse described a high-alert heparin episode in these terms: ‘I stopped the administration until we clarified the concentration; the dominant criterion was protocol, calculation and experience. If you start a wrong dose, you cannot easily repair it’ (A01). A physician in anaesthesia described a similar but more explicitly protocol-backed rule: ‘The alternative was to administer it because it was probably the right ampoule; that is exactly how errors happen. I did not accept it’ (M02). The contrast suggests that stop rules were strongest when local vigilance could be attached to a recognised procedural language.

### 3.2. Consent and Confidentiality Were Compressed Under Urgency, Exposure and Family Pressure

Participants drew a firm distinction between documentary completion and substantive understanding. A surgical resident stated, ‘The signature on its own is not enough’ (M01). That distinction became difficult to preserve in crowded wards, emergency transfers, labour, impaired capacity, and emotionally charged encounters with relatives. In urgent obstetric care, clinicians framed consent around the immediate reason for intervention and the major consequence of delay. One obstetrician described the target as ‘to inform enough for the immediate decision’ (M05). In emergency medicine and intensive care, diminished capacity shifted decisions towards vital interests, followed by later explanation to relatives where possible.

Confidentiality was constrained by the same organisational material. Corridors, shared rooms, multiple teams, and insistent relatives narrowed the conditions for private conversation. An emergency nurse remarked, ‘Confidentiality is almost impossible in the corridor’ (A03). Staff responded by lowering their voices, limiting detail, speaking to a single relative, or postponing fuller explanation until a more controlled moment. These practices reduced immediate informational leakage, yet they could generate suspicion, fragmented understanding, and later dispute about who had been told what.

Clinicians described this balancing act as a form of moral triage rather than as a rejection of ethical obligations. In high-pressure settings, they prioritised information needed for the immediate decision, preservation of life, or prevention of imminent harm, while postponing fuller explanation until the patient or relative could meaningfully receive it. One resident contrasted real consent with mere documentation: ‘Valid consent means that the patient understands the relevant risks and can say, in his or her own words, what will happen. The signature alone is not enough’ (M01). A nurse described the operational side of the same dilemma: confidentiality could be protected only by limiting details, lowering the voice, or delaying fuller disclosure until the corridor or shared room no longer controlled the conversation.

### 3.3. Voice and Reporting Depended on Legitimacy, Leadership, and Expected Cost

Speaking up was described as conditional rather than automatic. Staff weighed the clinical value of interruption against the social price of being seen as slow, difficult, or insubordinate. Protocol-backed risks such as high-alert medication, identification, and time-out procedures were easier to challenge because the interruption could be framed as standard practice rather than personal criticism. Organisational deficiencies were harder to raise. Broken equipment, fragile information technology, understaffing, and poor space design were frequently treated as ambient facts of work.

Formal reporting showed the same gradient. Across the 12 episodes, one incident was formally reported, one episode generated partial security-related documentation, and 10 episodes remained outside formal incident systems (Table 2; Supplementary Tables S2 and S8). Near misses were usually corrected locally, discussed verbally, and converted into small team rules. The gain was immediacy; the loss was organisational sight. Participants repeatedly linked silence to absent feedback, reputational risk, and the expectation that reporting would expose individuals without changing the process that generated the threat.

The single episode that entered the formal reporting system differed from most near misses because it was visible as an incident requiring observation, disclosure, or institutional traceability. By contrast, most near misses ended as locally corrected deviations: the patient was not harmed, the team restored the process, and the event was discussed verbally rather than converted into an organisational record. Participants therefore treated formal reporting as most likely when harm, family conflict, security involvement, or complaint risk made invisibility impossible.

Participants’ accounts also clarified the terms used in Table 2. In this study, a near miss denotes a potentially harmful event intercepted before patient harm occurred; an incident denotes an event that reached or affected the patient and created a need for observation, disclosure or formal traceability; a boundary event denotes a safety-critical episode at the edge between routine adaptation and reportable incident, where harm was not established but ethical or legal exposure shaped action; and a perceived incident denotes an episode framed by the patient, family, or staff as potentially incident-like even when formal classification remained uncertain.

The reporting interviews contained clear negative cases. A senior physician summarised the gap between ideal and real reporting as follows: ‘The ideal pathway is detection, reporting, analysis, feedback and change. In reality, we get stuck at feedback and change’ (M03). A nurse described the practical sequence as ‘detection, resolution in the team, sometimes telling the chief, rarely formal reporting, and almost never feedback’ (A01), while another participant described the form as a ‘black box’: ‘we complete it, give it to the chief, and then it disappears’ (A02). These quotations explain why only one episode became a formal report despite the presence of multiple learning opportunities.

### 3.4. Documentation and Disclosure Redistributed Accountability

Documentation served two overlapping functions throughout the material. It sustained continuity of care, and it allocated responsibility in anticipation of later scrutiny. Participants differentiated between notes that made clinical reasoning legible and notes that reduced exposure by narrowing what became sayable on the record. An internal medicine nurse recalled: ‘I documented “identification nonconformity, recollection repeated” without stating who switched the labels’ (A06). Similar patterns appeared when staff avoided naming near misses, omitted infrastructural weakness, or removed the interactional pressure that had shaped a decision.

This logic extended into communication with patients and relatives. When harm had not occurred, staff commonly said that treatment had been delayed, rechecked, or repeated ‘for safety’, while leaving the near miss itself unnamed. When visible harm or deterioration had occurred, disclosure moved towards factual control: what had happened, what had been done, and what would happen next. Participants viewed empathy and acknowledgement as clinically and ethically appropriate, yet many feared that explicit recognition of error would be read as a legal admission. The resulting style preserved immediate defensibility while making the underlying mechanism of danger harder for patients, relatives and the organisation to see.

A physician in anaesthesia made the mechanism of absence explicit: ‘I did not document that there had been ampoule confusion; the defensive component is precisely the absence—the near miss does not appear. The culture is that you write only what was done, not what you prevented’ (M02). In contrast, some entries were described as defensible because they made reasoning visible rather than because they concealed vulnerability. This distinction is important: documentation oriented to reasoning supported continuity, whereas documentation oriented to semantic minimisation protected the author while weakening organisational memory.

A nurse described the relational effect of defensive documentation after conflict: ‘after the incident everything moved to documents and who was to blame’ (A02). A physician described the same shift from another angle, noting that repeated standard phrases after complications could make records ‘less clinically useful and less sincere’ (M01). These nurse and physician accounts converged around a common mechanism: documentation could either preserve the context needed for learning or remove precisely the system weakness that made the event intelligible.

### 3.5. Cross-Case Pattern: Safety Work Became Fragile When Adaptation Stayed Local

**Figure 1:**
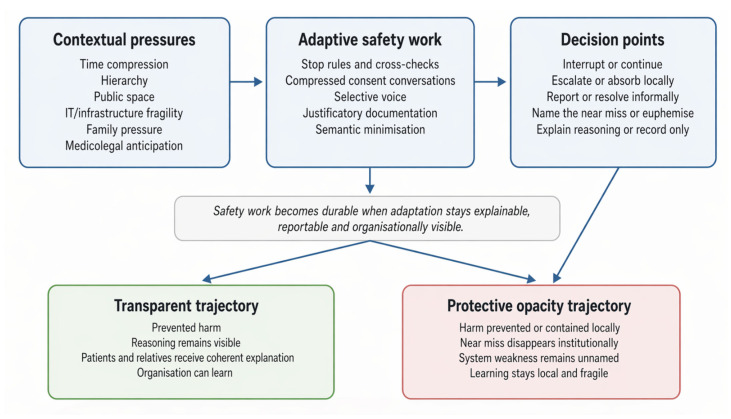
Integrative Conditions-Adaptations-Decisions-Consequences model of patient safety work at hospital interfaces. Organisational and relational pressures generate local adaptations that shape key decisions about interruption, escalation, documentation, and disclosure. When adaptations remain explainable and reportable, they support prevention and organisational learning; when they become euphemised or invisible, they preserve immediate defensibility while weakening institutional learning. Blue boxes represent the core process components of the model (contextual pressures, adaptive safety work, and decision points); the green box represents the transparent trajectory; and the red box represents the protective opacity trajectory. Arrows indicate the directional relationships between model components and outcomes.

The supplementary cross-case coding sharpened this pattern. Documentation or disclosure pressure was present in all 12 episodes, and voice or reporting considerations appeared in 10. Nurses more often narrated breakdowns in medication administration, specimen handling, and bedside identification; physicians more often centred urgent consent, inter-service escalation, and post-incident communication. Those differences did not produce separate worlds of safety. Rather, they showed that the same hospital system became visible through different interfaces. Safety held when local adaptation could interrupt the process and remain explainable. It became fragile when adaptation stayed local, euphemised, or detached from institutional learning (Table 3; Figure 1; Supplementary Tables S3–S11).

The mechanisms therefore interacted dynamically rather than operating as separate themes. A stop rule could prevent immediate harm; if the event was then described only as a delay or adjustment, the near miss disappeared from institutional memory; if the record also avoided the infrastructure, staffing, or communication weakness that made the near miss possible, the organisation lost the chance to redesign the interface. Conversely, when interruption was legitimised, reasoning recorded, and feedback returned to the team, the same local adaptation could move from individual rescue to organisational learning.

## 4. Discussion

### 4.1. Statement of Principal Findings

This study identified four interlocking mechanisms through which patient safety was produced and destabilised in everyday hospital work. Local stop rules created temporary stability at fragile interfaces; consent and confidentiality were compressed by urgency, public exposure, and family pressure; speaking up and reporting were regulated by legitimacy and leadership; and documentation and disclosure redistributed accountability between continuity of care and self-protection. The cross-case pattern suggests that hospital safety depends as much on what organisations can see and learn from as on what frontline staff manage to avert in the moment.

### 4.2. Strengths and Limitations

The study drew on deliberately varied episodes involving nurses and physicians from multiple high-risk settings. Incident-focused interviewing yielded process-rich accounts rather than broad attitudinal statements, and the analysis pursued mechanism across cases rather than theme alone. The supplementary cross-case coding and appended matrices extend the transparency of the analytic process.

Several limitations remain. The study was conducted in one public hospital, which limits transferability across legal and organisational contexts. The material is retrospective and therefore shaped by recall, interpretation, and presentation of self. Patients and relatives were outside the sample, which leaves the relational experience of consent, confidentiality, and disclosure only partly visible. Quotations were translated from Romanian into English, and some semantic nuance may have shifted in translation.

Two further limitations should be noted. First, the recruitment record did not retain a separate denominator of all staff invited or all refusals, which limits transparency about non-participation even though the completed sample was purposively varied. Second, the absence of patient and family interviews means that we cannot determine whether compressed consent was experienced as adequate, whether guarded disclosure protected or weakened trust, or whether relatives interpreted semantic minimisation as professionalism, uncertainty or avoidance.

### 4.3. Interpretation Within the Context of the Wider Literature

The findings align with resilient healthcare scholarship showing that safety is sustained through adaptive work at the boundary between formal procedure and practical contingency [3,4]. The present material extends that literature by demonstrating how adaptation acquires ethical and medicolegal weight at the moment of use. A delayed administration, shortened explanation, or strategically narrow note extends beyond operational adjustment; it reshapes autonomy, accountability, and the institution’s capacity to know what almost happened.

The findings on voice resonate with prior studies of speaking up in hierarchical clinical environments [8,9,17]. What this analysis adds is a sharp asymmetry between clinically codified risks and organisational deficiencies. Staff found it easier to interrupt a dose, label, or identity step than to challenge chronic understaffing, failing information systems, or defective infrastructure. This generated a pattern of local rescue without structural repair. Such a pattern is consistent with incident reporting literature showing that reporting becomes a poor bargain when exposure is personal and feedback scarce [10,11]. International work on safety governance has argued for learning architectures that take seriously both failure and success in frontline work [18]. The data from this study suggest that such architectures remain difficult to achieve when near misses are corrected competently yet disappear institutionally.

The Romanian context helps explain why medicolegal anticipation was so salient in the accounts. The national framework combines statutory patient rights to information, consent, and confidentiality [19], professional and institutional malpractice exposure under healthcare reform legislation [20], and accreditation-related expectations for adverse-event learning, including anonymised, non-accusatory reporting through the national adverse-event register promoted by ANMCS [21]. In practice, participants described a gap between this formal learning logic and the everyday fear that written traces might expose individuals or the institution before they produced visible improvement.

Corrected near misses are especially important in this context because they create a ‹‹#paradox of successful rescue. At the bedside, local correction protects the immediate patient and may appear to confirm that the system has recovered. Over time, however, repeated local correction without reporting can make latent conditions harder to see: missing barcode capacity, unreliable downtime procedures, understaffing, fragile handover fields, or lack of private communication space remain ambient rather than redesignable. Institutional blindness to corrected near misses therefore converts resilience into a hidden dependency on individual vigilance.

Some mechanisms are likely transferable beyond Romania: local checking at fragile interfaces, higher speaking-up thresholds in hierarchical teams, and under-reporting where feedback is weak have been reported internationally. Other features are more context-sensitive: limited privacy in crowded public hospital spaces, reliance on informal escalation relationships, fear of blame in a high-liability environment, and constrained infrastructure may intensify the tendency toward protective opacity.

The analysis of documentation and disclosure speaks to work on unsafe documentation, open disclosure, and just culture [12,13,22,23,24]. Participants distinguished between justificatory reasoning and protective opacity. The first made decisions auditable in context. The second protected the author more narrowly by suppressing mechanism, tension, or infrastructural weakness. That distinction has practical significance. Records that show reasoning can sustain continuity, collegial understanding, and defensible practice. Records that erase the route by which danger emerged preserve a cleaner document at the cost of poorer organisational memory. A similar tension shaped disclosure. Factual, tightly bounded communication held immediate conflict within manageable limits, yet it could leave patients and relatives with a residual sense that something material had been withheld.

### 4.4. Implications for Policy, Practice and Research

The most promising interventions target interfaces rather than individual vigilance. High-alert medication pathways, specimen labelling, patient identification during information technology downtime, and transfer documentation for time-critical medicines warrant explicit redundancy, narrow standardisation, and reliable backup systems. Urgent consent needs organisational recognition as clinical work rather than administrative residue: brief decision aids, protected consent slots for scheduled procedures, role allocation during crisis, and written prompts for post-event explanation would each address the gap between formal completion and substantive understanding.

Reporting systems require a psychologically safer route for near-miss reporting, rapid acknowledgement, and visible closure of the loop. Leadership conduct matters here because participants described psychological safety as locally produced. Training for senior clinicians and charge nurses should therefore include how to receive interruption, how to preserve legitimacy when juniors halt the flow, and how to communicate after incidents without driving the record towards euphemism. Future research would benefit from multisite comparison, direct inclusion of patients and relatives, and examination of how legal environment and hospital governance alter the threshold between transparent reasoning and protective opacity.

In resource-limited hospitals, the most feasible interventions are those that reduce ambiguity without requiring large capital investment. Examples include a one-minute protected near-miss reporting route with feedback within a defined timeframe, mandatory last-dose fields for time-critical transfers, a standard downtime identification protocol, explicit permission for nurses and junior physicians to stop high-risk processes, designation of a single family communication contact in crowded settings, and short prompts for urgent consent and post-event explanation. These measures operationalise transparency by making the safer action easier to perform, document, and learn from. The organisational leverage points identified through the cross-case analysis are summarised in Table 4.

## 5. Conclusions

Patient safety in hospital practice is assembled through situated judgement under organisational constraint, ethical pressure, and anticipated legal scrutiny. The clinicians in this study protected patients through local barriers, tactical delay, and selective escalation. The same accounts show how fragile that protection becomes when voice is costly, interfaces are weak, and records cannot safely name the mechanism of danger. Safer care depends on organisations that make transparent safety work easier to perform, document, and learn from. Practically, this means protecting near-miss reporting, closing feedback loops, legitimising psychologically safe escalation, and supporting urgent consent, confidentiality, and disclosure work with simple but explicit interface procedures.

## Figures and Tables

**Table 1:** Participant characteristics.

Code	Profession	Clinical Setting	Experience	Predominant Shift
A01	Nurse	ICU	12 years	Night/mixed
A02	Nurse	General surgery (postoperative ward)	7 years	Day (occasional on-call)
A03	Nurse	Emergency department	4 years	Mixed (day/night/weekend)
A04	Nurse	Paediatrics	10 years	Day (occasional mixed)
A05	Nurse	Oncology day care	6 years	Day
A06	Nurse	Internal medicine	15 years	Mixed (mostly nights)
M01	Resident physician	General surgery	2 years	Mixed (frequent on-call)
M02	Specialist physician	ICU/operating theatre (anaesthesia)	8 years	Mixed
M03	Senior physician	ICU	18 years	Day (with on-call)
M04	Specialist physician	Emergency department	5 years	Mixed
M05	Specialist physician	Obstetrics-Gynaecology	12 years	Mixed (on-call)
M06	Specialist physician	Internal medicine/Cardiology	9 years	Day (occasional on-call)

**Table 2:** Inventory of the 12 critical episodes and their reporting trajectories.

Code	Episode Type	Primary Safety Interface	Formal Reporting	Learning That Followed
A01	Near miss	High-alert medication	No	Proposal for standard notification of concentration changes
A02	Incident	Observation/disclosure	Yes	Brief discussion; no infrastructure change
A03	Near miss	Identification	No	Local reminder on two identifiers; no robust downtime backup
A04	Near miss	Medication and family mediation	No	Verbal lesson and suggestion for mandatory allergy field
A05	Near miss/boundary event	Procedural monitoring	No	Local rules reinforced; vascular access problem unchanged
A06	Near miss	Specimen identification	No	Stronger handover to laboratory; barcode absent
M01	Boundary event/perceived incident	Consent and post-incident communication	No	Document consent discussions more explicitly
M02	Near miss	High-alert medication	No	Local rule: no unlabelled syringe
M03	Boundary event	Time-critical treatment	No	Internal discussion; no staffing redesign
M04	Boundary event	Capacity, confidentiality and security	Partial	Improvised local practices; no stable protocol
M05	Boundary event	Urgent consent	No	Need for crisis communication role allocation
M06	Near miss	Transfer and medication timing	No	Local rule: no administration without time of last dose

**Table 3:** Recurrent mechanisms shaping patient safety work across cases.

Mechanism	Activating Conditions	Typical Adaptation	Immediate Gain	Longer-Term Cost
Interface stop rules	Mismatched labels, doses, identifiers, or transfer information	Pause, cross-check, call for confirmation, repeat collection, or delay administration	Prevents immediate harm at the bedside	Near misses may remain invisible to the organisation if the episode is corrected locally
Compressed consent and protected communication	Urgency, impaired capacity, crowded space, intense family pressure	Brief explanation focused on immediate decision; delayed fuller discussion; restricted disclosure to one relative	Preserves actionability under severe time and space constraint	Substantive understanding becomes fragile and later conflict becomes more likely
Hierarchy-conditioned voice and reporting	Strong gradients of authority, variable leader receptivity, reputational risk	Speak up through protocol language; selective silence about system defects; informal local debriefs	Makes interruption socially possible in some situations	Organisational threats remain chronic when staff avoid formal escalation
Documentation and disclosure under medicolegal anticipation	Conflict, complaint risk, punitive culture, absent disclosure support	Record the outcome and reasoning, yet omit tension, system weakness, or near-miss naming	Supports continuity and immediate defensibility	Protective opacity weakens institutional memory, patient trust, and system redesign

**Table 4:** Organisational leverage points emerging from the cross-case analysis.

Interface	Recurrent Failure Mode	Local Workaround Observed	System Redesign with Greatest Leverage
High-alert medication	Concentration changes, look-alike products, multitasking	Manual recalculation, self-imposed stop rule, discard and remake	Standard concentration alerts, labelling discipline, protected interruption for verification
Identification and specimen handling	Downtime, single printer, handwritten labels, interruptions	Two identifiers, relabelling, repeated collection, laboratory cross-check	Redundant printing or barcode-at-bedside, standard downtime protocol
Transfer and handover	Missing last-dose information, fragmented documentation, phone dependence	Call-back to sending ward, local rules, delayed administration	Mandatory transfer fields for time-critical medicines and interoperable records
Urgent consent	List pressure, labour pain, fear, impaired capacity	Compressed explanation, staged information, post-event clarification	Protected consent time for planned cases; crisis communication prompts for urgent care
Confidentiality in exposed space	Corridors, shared rooms, filming, multiple relatives	Minimal necessary information, single family contact, delayed fuller discussion	Dedicated spaces and role allocation for family communication
Near-miss learning and disclosure	Punitive expectations, absent feedback, fear of complaint	Verbal correction, semantic minimisation, guarded factual disclosure	Protected near-miss reporting, rapid feedback, structured disclosure support

## Data Availability

Because the dataset consists of de-identified qualitative interviews from a single institution, public sharing may create a risk of deductive disclosure. De-identified excerpts and the coding framework are available from the corresponding author on reasonable request, subject to ethics approval and institutional permissions.

## Supplementary Materials

The following supporting information can be downloaded at: https://www.mdpi.com/article/10.3390/healthcare14111542/s1, Online Supplementary Appendix (Tables S1–S11 and Figure S1), Table S1: Participant profile matrix; Table S2: Inventory of the 12 reconstructed critical episodes; Table S3: Episode-domain coding matrix used for descriptive cross-case display; Table S4: Full Conditions-Adaptations-Decisions-Consequences reconstruction across all 12 episodes; Table S5: Context-specific compression of consent and autonomy; Table S6: Confidentiality grey zones and protective compromises; Table S7: Speaking-up conditions, informal costs and safety effects; Table S8: Reporting, feedback and learning trajectories; Table S9: Documentation-disclosure continuum; Table S10: Profession-specific salience of coded domains; Table S11: Negative cases and analytic leverage; Figure S1: Cross-case domain coding across the 12 reconstructed critical episodes, plus the completed COREQ checklist, are provided as separate Supplementary Files.
