# Supplementary material for: Ethical, Medicolegal, and Organisational Pressures Shape Patient Safety at Hospital Interfaces: A Qualitative Study from Romania
Source: Healthcare (Basel). 2026 Jun 1;14(11):1542. doi: 10.3390/healthcare14111542 (PMC13257053; doi:10.3390/healthcare14111542)
Supplement: Supplementary file 1 [file healthcare-14-01542-s001.zip › healthcare-4317736-supplementary.pdf]

Online Supplementary Appendix

Ethical, medicolegal and organisational pressures shape patient safety at hospital interfaces: a qualitative study from Romania

Supplementary analytic note

This appendix makes explicit the cross-case descriptive coding used to support the analytic displays in the main manuscript. Coding was episode-based. Presence of a domain indicates that the domain was analytically salient within that critical episode; it does not indicate frequency, magnitude or causal priority. The supplementary material is organised to show how the explanation was built from the original critical-incident narratives into cross-case mechanism tables. Tables S1 to S11 and Figure S1 are intended for online publication and were kept separate from the blinded main manuscript in order to preserve compliance with the journal's limit on display items in the primary article.

Table S1. Participant profile matrix

Code	Profession	Clinical setting	Experience	Predominant shift
A01	Nurse	ICU	12 years	Night / mixed
A02	Nurse	General surgery (postoperative ward)	7 years	Day (occasional on-call)
A03	Nurse	Emergency department	4 years	Mixed (day/night/weekend)
A04	Nurse	Paediatrics	10 years	Day (occasional mixed)
A05	Nurse	Oncology day care	6 years	Day
A06	Nurse	Internal medicine	15 years	Mixed (mostly nights)
M01	Resident physician	General surgery	2 years	Mixed (frequent on-call)
M02	Specialist physician	ICU / operating theatre (anaesthesia)	8 years	Mixed
M03	Senior physician	ICU	18 years	Day (with on-call)
M04	Specialist physician	Emergency department	5 years	Mixed
M05	Specialist physician	Obstetrics-Gynaecology	12 years	Mixed (on-call)
M06	Specialist physician	Internal medicine / Cardiology	9 years	Day (occasional on-call)

Table S2. Inventory of the 12 reconstructed critical episodes

Code	Profession	Setting	Critical episode	Type	Primary interface	Formal reporting	Feedback	Learning outcome
A01	Nurse	ICU	Heparin infusion concentration change	Near miss	High-alert medication	No	Verbal only	Proposal for standard notification of concentration changes
A02	Nurse	General surgery	Postoperative patient fall	Incident	Observation/disclosure	Yes	Minimal	Brief discussion; no infrastructure change
A03	Nurse	Emergency department	Wrong-patient premedication avoided during wristband downtime	Near miss	Identification	No	None	Local reminder on two identifiers; no robust downtime backup
A04	Nurse	Paediatrics	Antibiotic withheld after allergy alert	Near miss	Medication and family mediation	No	None	Verbal lesson and suggestion for mandatory allergy field
A05	Nurse	Oncology day care	Early cytostatic extravasation detected	Near miss/boundary event	Procedural monitoring	No	Variable	Local rules reinforced; vascular access problem unchanged
A06	Nurse	Internal medicine	Blood samples mislabelled and withdrawn before analysis	Near miss	Specimen identification	No	Minimal	Stronger handover to laboratory; barcode absent
M01	Physician	General surgery	Compressed preoperative consent followed by postoperative complication	Boundary event/perceived incident	Consent and post-incident communication	No	Informal lesson only	Document consent discussions more explicitly
M02	Physician	Anaesthesia	Vial-syringe confusion avoided before administration	Near miss	High-alert medication	No	None	Local rule: no unlabelled syringe
M03	Physician	ICU	Antibiotic delay in sepsis under staffing pressure	Boundary event	Time-critical treatment	No	None	Internal discussion; no staffing redesign
M04	Physician	Emergency department	Altered patient with aggressive filming relatives	Boundary event	Capacity, confidentiality and security	Partial	Minimal	Improvised local practices; no stable protocol
M05	Physician	Obstetrics	Emergency caesarean with compressed consent	Boundary event	Urgent consent	No	None	Need for crisis communication role allocation
M06	Physician	Internal medicine/cardiology	Duplicate anticoagulant dose avoided at transfer	Near miss	Transfer and medication timing	No	None	Local rule: no administration without time of last dose

Table S3. Episode-domain coding matrix used for descriptive cross-case display

Code	Medication / procedure	Information / transfer	Consent / autonomy	Confidentiality / family	Voice / reporting	Documentation / disclosure
A01	Yes				Yes	Yes
A02				Yes		Yes
A03		Yes			Yes	Yes
A04	Yes			Yes	Yes	Yes
A05	Yes				Yes	Yes
A06		Yes			Yes	Yes
M01			Yes	Yes		Yes
M02	Yes				Yes	Yes
M03	Yes				Yes	Yes
M04			Yes	Yes	Yes	Yes
M05			Yes	Yes	Yes	Yes
M06	Yes	Yes			Yes	Yes

Table S4. Full Conditions-Adaptations-Decisions-Consequences reconstruction across all 12 episodes

Code	Conditions	Adaptation	Decision / action	Consequences
A01	Pharmacy syringe concentration changed while chart retained the routine dose expectation	Checked label, recalculated infusion rate, sought confirmation	Refused to start infusion until dose was clarified	Harm prevented; adjustment documented, near miss not named
A02	Postoperative patient with mobility risk, defective bed rail, family tension	Increased documentation after the event; selective description of supervision	Filed incident form yet omitted infrastructure weakness from the core narrative	Visible event entered the system; learning remained shallow
A03	Weekend crowding, wristband system failure, handwritten labels, similar patient names	Verbal identification and relabelling once the system recovered	Stopped administration and rebuilt the identification chain	Wrong-patient administration avoided; downtime remained institutionally underdescribed
A04	Paediatric antibiotic order in child with allergy history and family anxiety	Active questioning and interruption before administration	Withheld antibiotic and escalated allergy concern	Near miss prevented; lesson remained verbal and local
A05	Difficult venous access, irritant cytostatic, early subjective symptom	Immediate stop, aspiration, physician review, protocol activation	Treated mild symptoms as a threshold for emergency action	Tissue injury avoided; documentation became unusually detailed
A06	Single slow printer, interruptions, similar patient names, labels printed away from bedside	Laboratory cross-check and repeat collection	Withdrew samples before processing and recollected	Wrong result avoided; language became impersonal to reduce personal exposure
M01	Loaded operating list, anxious patient, public ward discussion, postoperative complication	Expanded record of explanations after conflict with relatives	Managed clinically, documented consent discussion more explicitly after complication	Complication handled; pressure from schedule and limited understanding disappeared from the record
M02	High-alert anaesthetic medication, vial-syringe resemblance, automation risk	Label-before-use discipline, discard and remake	Stopped immediately and rebuilt the syringe set-up	Catastrophic error prevented; near miss absent from formal records
M03	Time-critical sepsis treatment under staffing constraints and competing demands	Reasoned note explaining delay and prioritisation	Balanced urgency with available resources and documented the rationale	Care continued; staffing deficiency stayed largely invisible
M04	Altered patient, aggressive filming relatives, uncertain capacity, public emergency space	Security involvement, communication limitation, later clarification	Acted in vital interests while restricting disclosure until the situation stabilised	Patient and staff protected; documentation remained partial and improvised
M05	Foetal distress, minutes to decide, distressed patient, vocal partner	Compressed explanation centred on urgency and major consequences	Performed emergency caesarean and documented objective indicators of urgency	Maternal-fetal safety preserved; the consent conversation could not be fully captured
M06	Incomplete transfer record, unclear timing of last anticoagulant dose, warning from nurse/patient	Cross-ward verification and explicit note of rationale	Delayed administration until timing was confirmed	Duplicate dosing avoided; local rule created without formal reporting

Table S5. Context-specific compression of consent and autonomy

Clinical context	Conditions compressing autonomy	How consent operated in practice	Recurrent documentary trace	Perceived risk
ICU / emergency care	Sedation, confusion, severe instability, time-critical action	Vital-interest decisions followed by staged explanation	Capacity status, justification for action, timing of decision	Later contestation if the reasoning for capacity and urgency is thinly documented
Emergency department with aggression or filming	Public exposure, conflict, uncertain legal representative	Clinical action proceeds while the environment is controlled	Formal note justifies both treatment and restriction of communication	Escalating conflict and data-protection concerns
Emergency obstetrics	Very short decisional window, intense emotion, vocal relatives	Explanation focuses on why action is needed now and the major consequences of delay	Objective indicators of urgency and timing dominate the record	Perceived coercion and post-event complaint
Scheduled surgery	List pressure, public ward space, preoperative anxiety	Standard explanation with uneven checking of understanding	After complications, the record foregrounds that risks were explained	False reassurance from signature-centred consent
Oncology day care	Repeated forms, high informational load, treatment fatigue	Longitudinal explanation reinforced by nurses during treatment	High traceability and very detailed recording when reactions occur	Patients may sign without processing the information
Paediatrics	Proxy decision-making, family disagreement, refusal under stress	Legal representative verified; understanding negotiated with parent	Exact timing and person informed are recorded carefully	Persistent tension when parental refusal heightens risk for the child
Internal medicine / cardiology	Many forms, multimorbidity, repeated procedures	Consent becomes fragmented across encounters	Impersonal standard formulations dominate	Poor understanding of why a procedure or medicine was used

Table S6. Confidentiality grey zones and protective compromises

Context	Grey zone	Risk-minimising practice	Secondary effect
Crowded emergency corridor	Clinical discussion is audible to bystanders and relatives	General language, delayed fuller explanation, single family contact	Fragmented understanding and heightened mistrust
Emergency filming or aggression	Disclosure can become public while staff safety deteriorates	Request to stop filming, security support, limit information until stabilisation	Communication becomes adversarial and resource-intensive
ICU bays and multiple teams	Telephone requests and bedside discussions are easily overheard	Quiet voice, minimal necessary content, communication via designated physician	Relatives may perceive under-information
Shared ward rooms	Questions arise at the bedside in front of other patients	Move discussion where possible, restrict detail, redirect to treating physician	Reduced communicative quality and family frustration
Oncology infusion room	Sensitive prognostic content discussed in common space	Brief discussion at chairside, fuller conversation elsewhere	Time and space pressures persist
Paediatrics	Family members request information about a child from one another	Clarify who is authorised, separate conversations, protect the child from some content	Intra-family tension can intensify
Inter-unit transfer	Phone and messaging apps compete with formal channels	Data minimisation and deletion of temporary messages	Latent breach risk remains when institutional channels are weak

Table S7. Speaking-up conditions, informal costs and safety effects

Observed mechanism	Conditions activating it	How voice appears in practice	Informal cost	Safety effect
Protocol-backed legitimacy	Recognisable high-risk task with explicit rule	Stop-and-check phrasing, request for a label, identifier or confirmation	Short delay and local irritation	Strong barrier against immediate harm
Leader-dependent psychological safety	Senior clinician receptive or defensive	Voice expands under receptive leadership and contracts under irritation	Abrupt shift in how much juniors dare to say	High between-shift variability
Clinical-organisational asymmetry	Problem originates in staffing, space, infrastructure or IT	Clinical risk is voiced; system weakness is left implicit	Reputational risk and futility	Structural defects persist
Informal sanction	Strong hierarchy and heavy workload	Replies such as 'you are slowing us down' or 'you make trouble'	Self-censorship and selective battles	Risk normalisation through silence
Peripheral voice	Weak signal raised by patient, family or laboratory staff	Escalation based on a symptom, discrepancy or parental concern	Occasional stigma for 'listening too much'	Earlier detection of incipient risk
Just culture versus blame	Review focuses on process or on the person on duty	System-oriented discussion versus shaming	Protection versus fear	Determines whether voice becomes habitual

Table S8. Reporting, feedback and learning trajectories

Episode	Type	Formal report	What entered the record	What stayed out	Feedback	Learning trajectory
A01: Heparin infusion concentration change	Near miss	No	Dose adjustment and confirmation	Near-miss label and initial disagreement	Verbal only	Proposal for standard notification of concentration changes
A02: Postoperative patient fall	Incident	Yes	Fall and immediate clinical response	Defective bed rail and family tension	Minimal	Brief discussion; no infrastructure change
A03: Wrong-patient premedication avoided during wristband downtime	Near miss	No	Delay and relabelling	Near miss and downtime vulnerability	None	Local reminder on two identifiers; no robust downtime backup
A04: Antibiotic withheld after allergy alert	Near miss	No	Allergy note and drug withheld	Admission-process weakness	None	Verbal lesson and suggestion for mandatory allergy field
A05: Early cytostatic extravasation detected	Near miss/boundary event	No	Detailed treatment chronology	Language of error or blame	Variable	Local rules reinforced; vascular access problem unchanged
A06: Blood samples mislabelled and withdrawn before analysis	Near miss	No	Repeat collection after identification problem	Personal attribution of label switch	Minimal	Stronger handover to laboratory; barcode absent
M01: Compressed preoperative consent followed by postoperative complication	Boundary event/perceived incident	No	Clinical course, risks explained, relatives informed	Compressed initial discussion and schedule pressure	Informal lesson only	Document consent discussions more explicitly
M02: Vial-syringe confusion avoided before administration	Near miss	No	Only administered medications	Prevented near miss	None	Local rule: no unlabelled syringe
M03: Antibiotic delay in sepsis under staffing pressure	Boundary event	No	Clinical reasoning for delay	Direct naming of staffing deficit	None	Internal discussion; no staffing redesign
M04: Altered patient with aggressive filming relatives	Boundary event	Partial	Mental status and security issue	Full relational texture of aggression	Minimal	Improvised local practices; no stable protocol
M05: Emergency caesarean with compressed consent	Boundary event	No	Timing, urgency, CTG and intervention	Full content of compressed consent conversation	None	Need for crisis communication role allocation
M06: Duplicate anticoagulant dose avoided at transfer	Near miss	No	Clarification of last dose and rationale for delay	Inter-service friction	None	Local rule: no administration without time of last dose

Table S9. Documentation-disclosure continuum

Continuum position	Documentation pattern	Disclosure pattern	Typical trigger	Likely consequence
Transparent reasoning	Decision, uncertainty and rationale are recorded explicitly	Patients and relatives receive factual explanation plus plan and follow-up	Supportive leadership, clear protocol, psychologically safer climate	Continuity, trust and usable learning
Reasoned defensibility	Context and logic are recorded, yet system pressures are softened	Communication remains factual and bounded	Fear of complaint with some confidence in internal review	Adequate continuity with partial loss of organisational detail
Selective opacity	Outcome is recorded while the route into danger is narrowed	Near miss is described as delay, recheck or repetition	Punitive culture, weak reporting value, absent disclosure framework	Near miss disappears and system memory thins
Protective impersonalism	Passive voice and generic terms replace personal or structural attribution	Relational content and acknowledgement are reduced	Strong exposure anxiety and expectation of blame	Immediate self-protection with poorer trust and weaker redesign

Table S10. Profession-specific salience of coded domains

Profession	Episodes (n)	Medication / procedure	Information / transfer	Consent / autonomy	Confidentiality / family	Voice / reporting	Documentation / disclosure	Interpretive note
Nurse	6	3	2	0	2	5	6	Episodes concentrated on bedside execution and material interfaces.
Physician	6	3	1	3	3	5	6	Episodes concentrated on urgent decision-making, escalation and post-event communication.

COREQ Checklist

No.	Item	Guide question / description	Reported on page(s)	Notes
**Domain 1: Research team and reflexivity**				
1	Interviewer/facilitator	Which author conducted the interview/focus group?	1	Reported as 'a physician in residency training' conducting the interviews.
2	Credentials	Researcher's credentials (e.g., PhD, MD)	1	Partly reported: physician in residency training with university affiliation; formal degree not fully specified.
3	Occupation	Researcher's occupation at the time of the study	4	Reported.
4	Gender	Was the researcher male or female?	Not reported	Add before submission if the authors wish full COREQ coverage.
5	Experience and training	What experience or training did the researcher have?	Not reported	Qualitative interviewing training is not specified.
6	Relationship established	Was a relationship established prior to study commencement?	4	Partly addressed: no direct supervisory relationship reported; prior relationship otherwise not specified.
7	Participant knowledge of the interviewer	What did participants know about the researcher?	4	Partly addressed: recruitment/interviewing were framed as non-evaluative.
8	Interviewer characteristics	Biases, assumptions, reasons or interests in the topic	5	Partly addressed through reflexive memos and analytic stance; interviewer-specific assumptions not detailed.
**Domain 2: Study design**				
9	Methodological orientation and theory	Methodological orientation/theory underpinning the study	3-5	Critical realist orientation and framework method reported.
10	Sampling	How were participants selected?	3	Purposive maximum-variation sampling reported.
11	Method of approach	How were participants approached?	4	Partly reported: neutral invitations and individual scheduling are described; communication channel is not specified.
12	Sample size	How many participants were in the study?	3	Twelve clinicians.
13	Non-participation	Refusals/dropouts and reasons	Not reported	Add if available.
14	Setting of data collection	Where was the data collected?	3-4	Public hospital in Romania and included clinical areas reported.
15	Presence of non-participants	Was anyone else present besides researchers/participants?	Not reported	Add if relevant.
16	Description of sample	Important participant characteristics	3-4	Reported in text and Table 1.
17	Interview guide	Were questions/prompts/guides provided? Pilot tested?	4	Guide domains reported; pilot testing not reported.
18	Repeat interviews	Were repeat interviews carried out?	4	Reported: each participant took part in one interview.
19	Audio/visual recording	Was recording used to collect data?	4	Audio-recording reported.
20	Field notes	Were field notes made during/after the interview?	5	Partly addressed through reflexive memos after interviews.
21	Duration	Duration of interviews/focus groups	Not reported	Add if available.

No.	Item	Guide question / description	Reported on page(s)	Notes
22	Data saturation	Was data saturation discussed?	3	Recruitment stopping rule is reported.
23	Transcripts returned	Were transcripts returned for comment/correction?	Not reported	Add if applicable.
**Domain 3: Analysis and findings**				
24	Number of data coders	How many data coders coded the data?	Not reported	Add if available.
25	Description of the coding tree	Was a coding tree/framework described?	4-5	Analytic structure, matrices and Conditions-Adaptations-Decisions-Consequences logic reported.
26	Derivation of themes	Were themes identified in advance or derived from the data?	4-5	Both deductive and inductive components reported.
27	Software	Software used to manage the data	Not reported	Add if applicable.
28	Participant checking	Did participants provide feedback on findings?	Not reported	Add if applicable.
29	Quotations presented	Were participant quotations presented and identified?	5-8	Yes; quotations are labelled with participant codes.
30	Data and findings consistent	Consistency between data presented and findings	5-12	Findings are supported by text, quotations, tables and integrative figure.
31	Clarity of major themes	Were major themes clearly presented?	5-12	Yes.
32	Clarity of minor themes	Were diverse/minor cases described?	5-11	Negative cases and cross-case variation are explicitly discussed.

Table S11. Negative cases and analytic leverage

Negative or divergent case	Why it mattered analytically	What it refined in the interpretation
Highly standardised oncology documentation (A05)	Detailed recording did not always indicate openness; meticulous notes could coexist with avoidance of error language	Documentation quality and transparency are related yet not identical
Senior physician note explaining delay in sepsis (M03)	A defensive motive did not automatically produce opacity; reasoning could be recorded in a clinically useful way	Defensiveness has a spectrum ranging from transparent justification to concealment
Emergency obstetric consent with good clinical outcome (M05)	Compression of consent did not inevitably lead to complaint or poor care when urgency was clear and objective traces were strong	Consent quality depends on context, sequence and later explanation, not on duration alone
Laboratory detection of specimen mismatch (A06)	Safety work extended beyond the originating unit and depended on downstream actors	Organisational learning needs to include cross-departmental barriers, not only bedside behaviour

Figure S1. Cross-case domain coding across the 12 reconstructed critical episodes

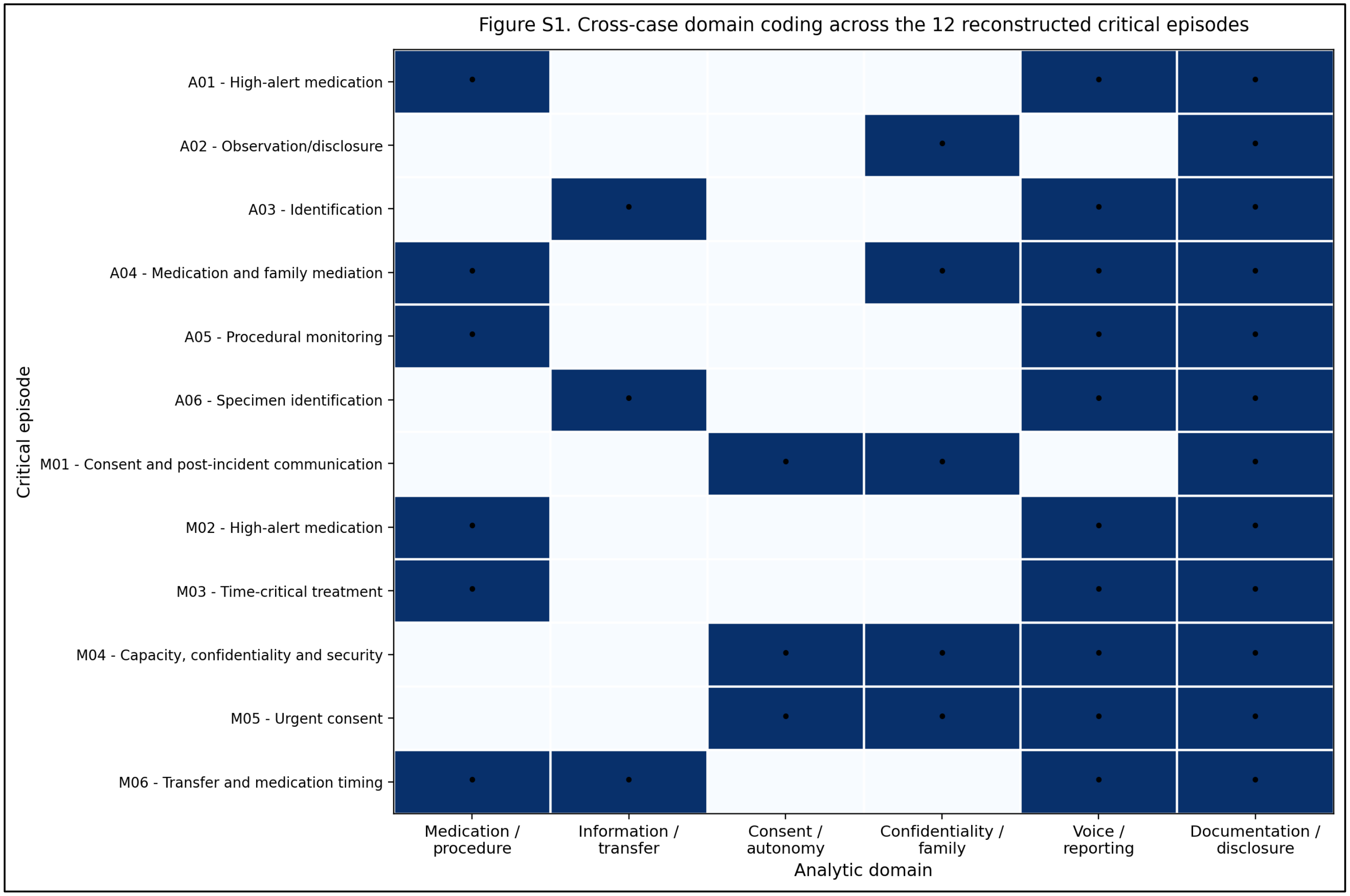

Filled cells indicate that the domain was analytically salient within the episode.
